# Ectopic expression of cyclase associated protein CAP restores the streaming and aggregation defects of adenylyl cyclase a deficient *Dictyostelium discoideum *cells

**DOI:** 10.1186/1471-213X-12-3

**Published:** 2012-01-12

**Authors:** Hameeda Sultana, Girish Neelakanta, Francisco Rivero, Rosemarie Blau-Wasser, Michael Schleicher, Angelika A Noegel

**Affiliations:** 1 Center for Biochemistry, Medical Faculty, University of Cologne, 50931 Köln, Germany; 2 Section of Infectious Diseases, Department of Internal Medicine, Yale University School of Medicine, 300 Cedar Street, New Haven, CT 06520, USA; 3 Centre for Cardiovascular and Metabolic Research, The Hull York Medical School and Department of Biological Sciences, University of Hull, Hull HU6 7RX, UK; 4 Institute for Anatomy and Cell Biology, Ludwig-Maximilians-Universität, 80336 München, Germany; 5 Center for Molecular Medicine Cologne (CMMC), University of Cologne, 50931 Köln, Germany; 6 Cologne Excellence Cluster on Cellular Stress Responses in Aging-Associated Diseases (CECAD), University of Cologne, 50931 Köln, Germany

**Keywords:** Cell polarity, aggregation, cell-adhesion, development, Cyclase associated protein, Adenylyl cyclase ACA, Adenylyl cyclase ACB

## Abstract

**Background:**

Cell adhesion, an integral part of *D. discoideum *development, is important for morphogenesis and regulated gene expression in the multicellular context and is required to trigger cell-differentiation. G-protein linked adenylyl cyclase pathways are crucially involved and a mutant lacking the aggregation specific adenylyl cyclase ACA does not undergo multicellular development.

**Results:**

Here, we have investigated the role of cyclase-associated protein (CAP), an important regulator of cell polarity and F-actin/G-actin ratio in the *aca^- ^*mutant. We show that ectopic expression of GFP-CAP improves cell polarization, streaming and aggregation in *aca^- ^*cells, but it fails to completely restore development. Our studies indicate a requirement of CAP in the ACA dependent signal transduction for progression of the development of unicellular amoebae into multicellular structures. The reduced expression of the cell adhesion molecule DdCAD1 together with csA is responsible for the defects in *aca^- ^*cells to initiate multicellular development. Early development was restored by the expression of GFP-CAP that enhanced the DdCAD1 transcript levels and to a lesser extent the csA mRNA levels.

**Conclusions:**

Collectively, our data shows a novel role of CAP in regulating cell adhesion mechanisms during development that might be envisioned to unravel the functions of mammalian CAP during animal embryogenesis.

## Background

The amoeba *Dictyostelium discoideum *has adopted a unique strategy for multicellular development, as in their vegetative stage, single-celled amoebae feed on bacteria and multiply by binary fission. Upon starvation, they embark on a developmental cycle where individual cells come together in response to the chemoattractant cAMP finally forming fruiting bodies that are highly differentiated multicellular structures [1,2]. These features of *D. discoideum *make it a valuable and convenient experimental model for studies related to signal transduction, cell migration, chemotaxis, cell adhesion, differentiation and development. Its development displays many features that are characteristics of mammalian embryogenesis including the tightly regulated cell-cell adhesion. Cell adhesion, an integral part of *D. discoideum *development, is important for morphogenesis and regulated gene expression in the multicellular context and is required to trigger cell-differentiation [3-5].

Specific cell-cell adhesion mechanisms are involved in maintaining the integrity and stability of the cell aggregates, and at least three different types of cell-cell adhesion sites are expressed during *D. discoideum *development. During early development, two glycoproteins, *D*. *discoideum *cadherin 1, DdCAD1 (gp24), and contact site A, csA (gp80), mediate cell-cell adhesion between amoebae as they form loosely packed multicellular structures [5-7]. DdCAD1 mediated cell adhesion is sensitive to both EDTA and EGTA, suggesting that Ca^2+ ^is involved in the process, however, csA-mediated adhesion is Ca^2+^-independent and insensitive to both EDTA and EGTA [6,8]. Soon after the initiation of starvation, DdCAD1 is enriched on the plasma membrane and as aggregation proceeds it reaches to the external surface of the plasma membrane. During aggregation, csA expression is dependent on DdCAD1, as when DdCAD1-mediated adhesion is blocked by EDTA, csA expression is severely reduced and even stimulation with cAMP failed to restore csA expression indicating that DdCAD1 mediated cell adhesion is required for full induction of csA. LagC/gp150 is expressed in cells in the early post-aggregation stage mediating a Ca^2+^-independent adhesion system [9,10].

cAMP signaling is crucial for the chemotactic aggregation of single cells into multicellular structures and for the succession through late development [2,11]. Aggregation centers secrete cAMP in pulses which are detected, amplified and relayed to the surrounding cells that sense this extracellular cAMP through their G-protein coupled cAMP receptors (cARs) located on the cell surface [12]. Activation of cAR1 receptors causes a dissociation of the G-proteins. The Gβγ complex together with the cytosolic regulator of adenylyl cyclase (CRAC) and Pianissimo activate aggregation specific adenylyl cyclase (ACA) that leads to the synthesis of extracellular cAMP and initiates the cAMP relay response [11]. Receptor mediated G-protein linked adenylyl cyclases are universal signal transducers that play important roles in signaling, leading to the directed migration of cells and development. Other than ACA, the homolog to the G-protein regulated mammalian adenylyl cyclase, *D. discoideum *harbors two more adenylyl cyclases: a germination specific adenylyl cyclase (ACG), expressed in prespore cells and spores and acting as osmosensor; and a G-protein independent adenylyl cyclase ACB harbouring histidine kinase and response-regulator domains that is required for terminal differentiation [13].

Although ACA is not required for chemotaxis, it is essential for the cells to align in a head to tail fashion and stream into aggregates, where ACA enriches at the uropod of the chemotaxing cells. This distribution of ACA is dependent on the proper regulation of the actin cytoskeleton and on the acquisition of cellular polarity. Cells lacking ACA are capable of moving up the chemoattractant gradient but are unable to stream and polarize thus exhibiting severe defects in cell polarity and aggregation [14]. During growth, *D. discoideum *cells display variable polarity by constantly changing their shape and forming new ends in response to the environmental cues that ultimately facilitates to target the food source. However, after the onset of starvation, periodic signals of cAMP lead to polarization of the cells and initiate development. Localized assembly of signaling complexes, directed cytoskeletal rearrangements and distinct recruitment of proteins are essential components of cellular polarity [15].

Cyclase associated protein (CAP), a regulator of the F-actin/G-actin ratio, has been identified as an important regulator of cell polarity in *D. discoideum *[16-19]. We have previously shown that a CAP mutant (CAP bsr) has severe defects in cell polarization that were also accompanied by reduced sensitivity to chemoattractant and altered cAMP relay response. Also, the cAMP induced cGMP response and the signal transduction pathways leading to chemotactically induced cell polarization were altered in CAP bsr cells [18]. In yeast, a short but highly conserved N-terminal stretch of CAP had been shown to physically interact with the C-terminus of adenylyl cyclase [20]. In spite of these highly conserved domains we were unable to detect any direct interaction between CAP and the aggregation specific adenylyl cyclase (ACA) in *D. discoideum*.

So far a direct link between CAP and adenylyl cyclase from organisms other than yeast has not been made. Here, we show a genetic interaction between ACA and CAP and demonstrate that ectopic expression of CAP restores cell polarity, streaming and aggregation defects of *aca^- ^*cells and elucidate the mechanism of signaling crosstalk between these proteins during *D. discoideum *development.

## Results

### Expression and distribution of GFP fusions of CAP and its domains in *aca^-^* cells

**Figure 1:**
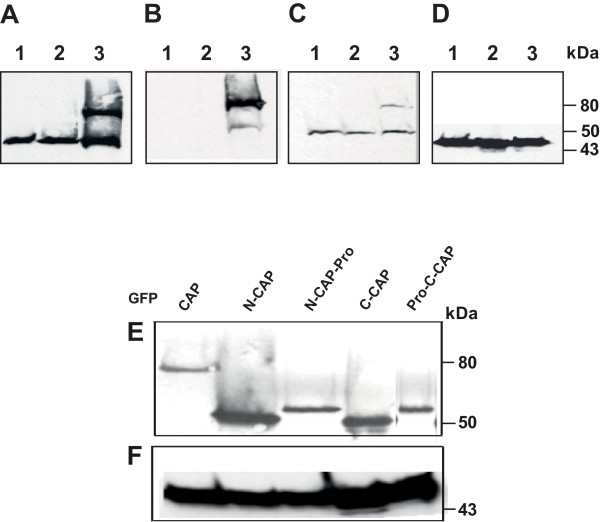
**Expression of CAP and GFP fusions of CAP or its domains in *aca^-^* cells**. 5 × 10^5 ^cells were lysed in 2 × SDS sample buffer and separated on 12% SDS-PAGE. Immunoblots show the expression of endogenous CAP and GFP-fusions of full length or N- or C-terminal domains of CAP with or without the proline rich regions in *aca^-^* cells. Endogenous CAP (50 kDa) and GFP-CAP (~80 kDa) were detected with mAb 230-18-8 (A) or with GFP specific mAb K3-184-2 (B) that detected only GFP-tagged proteins or with CAP C-terminus specific mAb 223-445-1 (C), that detected both endogenous and GFP-CAP. The lower band in B (*aca^-^* -GFP-CAP) is a degradation product. (D) Actin (~42 kDa) detected with mAb act1 served as loading control. (E) GFP-fusions of CAP in *aca^-^* cells were detected with mAb K3-184-2. (F), actin control. In A-D an AX2 lysate was included to allow the comparison for endogenous CAP in the wild type. A-D, lanes 1, AX2 whole cell lysate; lanes 2, *aca^-^* whole cell lysate; lanes 3, whole cell lysate from *aca^-^* cells expressing full length GFP-tagged CAP.

In CAP mutant cells normal ACA activity was measured when cells were presented with appropriate cAMP signals, however, there was less of an increase in ACA activity during development in unstimulated CAP mutant cells [18]. Here, we investigated the expression levels of endogenous and GFP fusions of CAP and its domains in unstimulated *aca^-^* cells. Transcription is under the control of the actin15 promoter allowing protein expression during all stages of *D. discoideum *growth and development. In *aca^-^* cells, endogenous CAP levels were comparable to the wild type control AX2 and GFP-CAP expression had no effect on the levels of endogenous CAP (Figure 1A-D). GFP-fusions of the different domains of CAP were also stably expressed in *aca^-^* cells, suggesting that CAP expression is not affected by the deficiency of ACA (Figure 1C, F).

Next, we determined the localization of GFP fusions of CAP and its domains in resting *aca^-^*cells and the redistribution during macropinocytosis and phagocytosis. Full length GFP-CAP distributed throughout the cytoplasm with enrichment close to the membrane and at fronts. GFP-N-CAP and GFP-N-pro-CAP localized throughout the cell and showed particular enrichment at front regions and in the periphery of the *aca^- ^*cells. GFP-C-CAP and GFP-Pro-C-CAP were present throughout the cells, however they were absent from the cell cortex (Additional file 1, Figure S1). Similar distributions had been previously reported for the GFP fusions of CAP and its domains in AX2 cells [17]. Our data suggest that ACA is not required for the correct targeting of CAP or its domains. Having established the correct localization of GFP fusions of CAP in *aca^-^* cells we went on to analyze if ACA is involved in CAP redistribution during membrane associated events such as macropinocytosis and phagocytosis using live cell imaging and observed a quick redistribution of GFP-CAP to macropinocytic cups and macropinosomes (Additional file 2, Figure S2A). Immunofluorescence studies in *aca^-^* cells revealed that both endogenous and GFP-CAP were prominently redistributed and localized to the regions of fluid uptake, and the N-terminal fusion of CAP also redistributed correctly to regions of pinocytic cup formations and pinosomes during macropinocytosis (Additional file 2, Figure S2B). During phagocytosis both endogenous and GFP-CAP redistributed to the sites of yeast engulfment forming phagocytic cups and phagosomes. GFP-N-CAP and GFP-N-pro-CAP behaved like GFP-CAP, whereas GFP-fusions of C-CAP neither were enriched nor showed an altered distribution during phagocytosis (Additional file 3, Figure S3A). In quantitative analysis we found no significant differences in yeast internalization for *aca^-^*transformants when compared to AX2 which suggested that ACA is not crucial during macropinocytosis or phagocytosis and expression of CAP (or its domains) did not interfere (Additional file 3, Figure S3B).

### Ectopic expression of CAP corrects the polarization defects of *aca^-^* cells

A previous report has shown that *aca^-^* cells display severe defects in polarity, remain virtually immobile and are incapable of generating streams thereby exhibiting aggregation defects [14]. CAP is also required for cell polarization, because the CAP bsr cells showed a delay in aggregation, were more rounded and did not exhibit the typical elongated shapes within these aggregates. Expression of GFP-CAP rescued these defects of CAP bsr cells [18]. Here, we have investigated if expression of GFP-CAP restores the polarity defect of *aca^-^* cells as well and studied the distribution of the cytoskeletal components myosin, α-actinin and filamin. In aggregation competent AX2 and AX2-GFP-CAP cells, myosin was redistributed to the rear ends and lateral sides of highly polarized cells. This localization is thought to suppress the formation of lateral pseudopods during cell migration [21]. α-actinin, an actin filament cross-linking protein, was present throughout the cytosol with enrichments at the leading fronts. Filamin, another F-actin crosslinking protein, distributed more prominently at the cell posterior with discontinuity at the leading edges of AX2 and AX2 cells expressing GFP-CAP, whereas in *aca^-^* cells the staining pattern was as in vegetative cells as they stayed more rounded (Additional file 4, Figure S4 and Additional file 5, Figure S5). In *aca^-^* cells expressing GFP-CAP the cells became more elongated and the distribution of the proteins was comparable to AX2 and AX2 expressing GFP-CAP (Additional file 6, Figure S6A). An altered cell shape and a corresponding distribution of polarity markers were also noted for *aca^-^* cells expressing GFP-N-pro-CAP (Additional file 6, Figure S6B). These data suggest that expression of GFP-CAP rescues the polarity defect and further, that the expression of the N-terminal domain of CAP is sufficient to restore the polarization defects of *aca^-^* cells.

### Expression of GFP-CAP restores the streaming and aggregation defects of *aca^-^* cells

**Figure 2:**
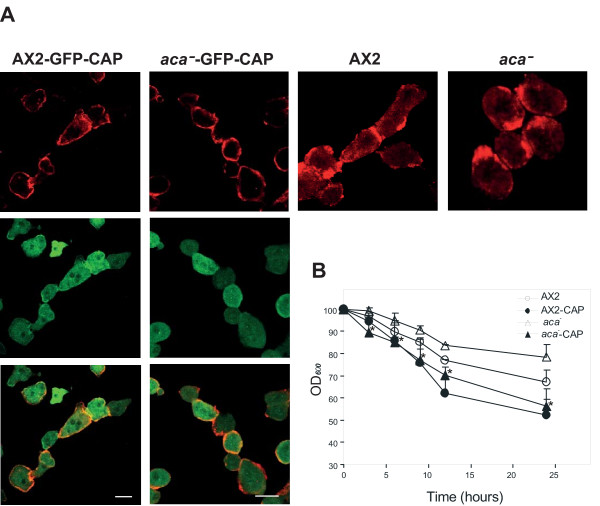
**Ectopic expression of GFP-CAP restores aggregation defects of *aca^-^* cells**. (A) Aggregation competent AX2 and *aca^-^*cells and corresponding transformants expressing GFP-CAP were starved for 6 h in Soerensen phosphate buffer. Cells were fixed with methanol at -20°C and immunostained with mAb act1 specific for actin. Bar, 10 μm. (B) Time course of agglutination. 1 × 10^7 ^cells/ml were starved in Soerensen phosphate buffer, and at the indicated times the optical density (OD) as a measure for cell agglutination was determined at 600 nm using a spectrophotometer. Error bars represent the standard deviation. Statistics were performed using the Student's t-test and the P value < 0.05 is indicated with *.

Cells lacking ACA fail to aggregate and remain as a homogenous monolayer indefinitely while the parental strain aggregates within 3 h of starvation and forms multicellular fruiting bodies by ~24 h [22]. To gain insights into the role of CAP during cell polarity and development, we examined the streaming and aggregation of *aca^-^* expressing GFP-CAP and in particular their ability to attach to each other end to end and to form chains of cells. In the contact regions actin and associated proteins are enriched. For AX2 we saw chains of cells at the 6 h time point in which the cells adhered to each other. In the contact zones actin was enriched. A similar behavior was also seen in AX2 cells expressing GFP-CAP. ACA-deficient cells at the same time point remained rounded. Upon expression of GFP-CAP the cells formed chains and in the contact regions actin was present indicating that CAP has an effect on cell polarity of *aca^-^*, however, the cells were less elongated than AX2 at the same time point (Figure 2A).

Quantitative analysis showed a significant increase in agglutination of starving *aca^-^* cells expressing GFP-CAP that was comparable to the agglutination of AX2. Expression of GFP-CAP in AX2 also enhanced agglutination suggesting an aggregation promoting effect of CAP (Figure 2B). To address which domain of CAP corrected the aggregation defects of *aca^-^* cells, we performed immunofluorescence studies with aggregation competent cells expressing N- or C-terminal CAP fusions with or without the proline rich regions. GFP-N-pro-CAP expression led to an increase in the stream and mound formation of *aca^-^* cells, whereas the C-terminal domain fusions showed fewer and smaller mounds (data not shown). Our data demonstrate that ectopically expressed GFP-CAP or GFP-N-pro-CAP ameliorates the polarity and streaming defects of *aca^-^* cells.

**Figure 3:**
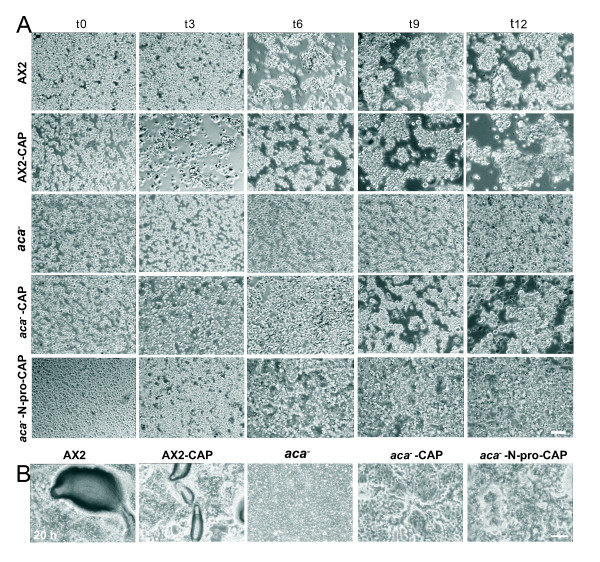
**Expression of GFP-CAP or GFP-N-pro-CAP restores aggregation defects but not complete development in *aca^-^* cells**. (A) Phase contrast microscopy showing aggregate formation of starving AX2, AX2 expressing GFP-CAP (AX2-CAP), *aca^-^* cells expressing GFP-CAP (*aca^-^*-CAP) or GFP-N-pro-CAP (*aca^-^*-N-pro-CAP). 1 × 10^7 ^cells/ml resuspended in Soerensen phosphate buffer were starved in suspension and images were obtained with 20x bright field phase contrast microscope at indicated times (in hours, h). (B) *aca^-^* cells and cells expressing GFP-CAP or GFP-N-pro-CAP were plated on phosphate agar plates and allowed to undergo development and fruiting body formation. Images were obtained with 20x bright field phase contrast microscope at 20 h of development. The *aca^-^* cells remained as single cells whereas expression of GFP-CAP or GFP-N-pro-CAP in* aca^-^* cells led to formation of aggregation streams and mounds which however failed to proceed into late developmental stages and fruiting body formation in comparison to AX2 and AX2 expressing GFP-CAP. The larger aggregate size observed for AX2 expressing GFP-CAP is probably due to higher levels of contact site A protein as determined by western blot analysis (Figure 4A). The multicellular structure shown for AX2 is quite large. It might have resulted from a higher cell density in this area of the plate. Bars, 100 μm in both (A) and (B).

**Figure 4:**
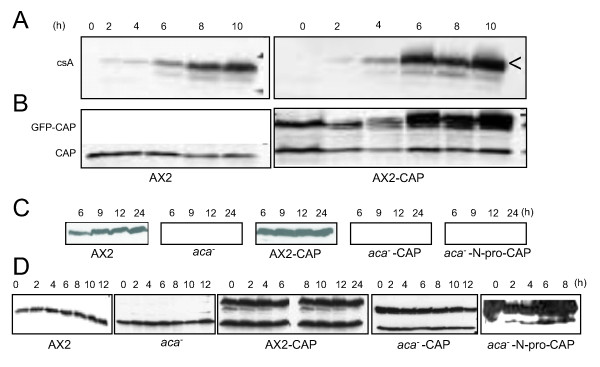
**Ectopic expression of GFP-CAP influences csA protein levels in AX2 but not in *aca^-^* cells**. Aggregation competent cells at a density of 1 × 10^7^/ml were starved in Soerensen phosphate buffer. Samples were collected at the indicated times (hours, h) and lysed in 2 × SDS sample lysis buffer and resolved by 10% SDS-PAGE. Western blots were probed with csA specific mAb 33-294-17 (A) or with CAP specific antibody mAb 230-18-8 (B) as indicated. Expression of csA was enhanced in AX2 cells expressing GFP-CAP when compared to AX2. Arrow indicates the mature 80 kDa form of csA, the band below is a precursor. In *aca^-^* cells or transformants expressing GFP-CAP or GFP-N-pro-CAP no csA expression was detected at the protein level (C). Blots were reprobed with CAP antibody to reveal CAP and GFP-CAP fusions as indicated (D).

We then analyzed aggregation competent *aca^-^* cells expressing GFP-CAP or GFP-N-pro-CAP for their ability to form agglutinates at different time points during development. Phase contrast microscopic images showed that wild type AX2 cells had agglutinated at 6 h of starvation whereas AX2 cells expressing GFP-CAP agglutinate before 6 h and form larger aggregates at later time points (Figure 3A). This is presumably due to higher levels of contact site A protein (see below, Figure 4). The *aca^-^* cells remained as single cells; however expression of GFP-CAP or GFP-N-pro-CAP restored the aggregation defect and led to formation of agglutinates albeit with a delay. The rescue potential of N-pro-CAP was not as high as the one of full length CAP (Figure 3A).

Aggregates can form under submerged conditions, whereas post aggregative development requires a solid substratum. When we analyzed development on phosphate agar plates, AX2 and AX2 cells expressing GFP-CAP formed multicellular structures (slugs and culminates) at 20 h, whereas the *aca^-^* cells remained as cell layers (Figure 3B). Upon starvation *aca^-^* cells expressing GFP-CAP or GFP-N-pro-CAP showed enhanced streaming (characteristic star-like patterns) and formed aggregates, but failed to undergo further development into slugs and fruiting bodies (Figure 3B). These data show that ectopic expression of CAP or its N-terminal domain while correcting streaming, polarity and early aggregation defects of *aca^-^* cells it does not restore development completely.

### GFP-CAP expression primarily mediates EDTA sensitive cell adhesion mechanisms in *aca^-^* cells

**Figure 5:**
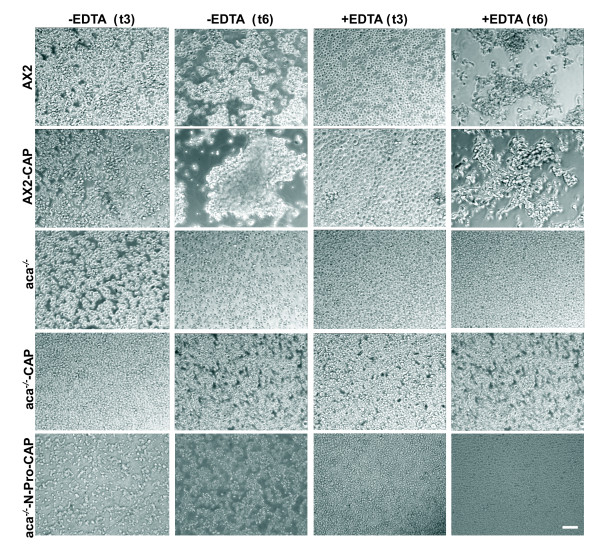
**Expression of GFP-CAP or GFP-N-pro-CAP in *aca^-^* cells promotes EDTA sensitive but not EDTA resistant cell adhesion**. Phase contrast microscopy showing cell-cell adhesion of AX2 and *aca^-^* cells and AX2 and *aca^-^* null cells expressing GFP-CAP or GFP-N-pro-CAP in the presence or absence of 10 mM EDTA. 1 × 10^7 ^cells/ml were incubated with agitation (160 rpm/min) at 21°C. Samples were collected at indicated time points (in hours, h) and incubated in the presence or absence of EDTA for an additional hour. Images were obtained with a 20x bright field phase contrast microscope. White arrow points to small aggregates in *aca^-^* cells expressing GFP-CAP in the presence of EDTA. Bar, 100 μm.

**Figure 6:**
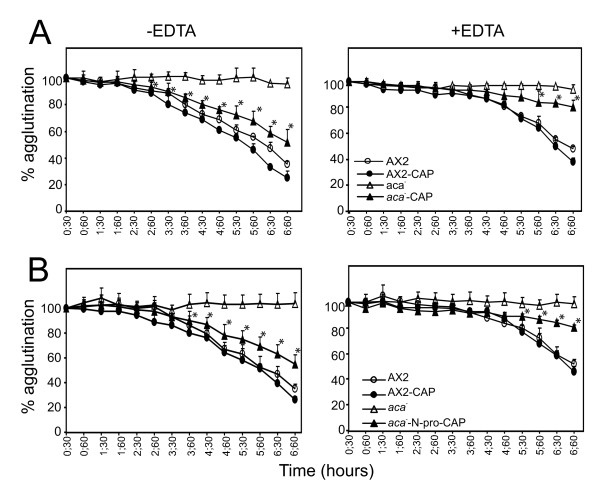
**Quantitative agglutination assays of AX2 and *aca^-^* cells expressing GFP-CAP or GFP-N-pro-CAP**. 1 × 10^7 ^cells/ml were starved in Soerensen phosphate buffer, collected at indicated times and incubated for an additional hour in the presence or absence of 10 mM EDTA. The OD600 was determined to measure the decrease in single cells as agglutination progresses in the presence or absence of EDTA. Agglutination is shown as percentages of the starting point (set to 100% which corresponds to essentially single cells in suspension). A decrease indicates aggregate formation. Agglutination of *aca^-^* cells expressing GFP-CAP (A) or GFP-N-pro-CAP (B) in the absence and presence of EDTA was significantly improved. Data represents the mean of three independent experiments. Bars represent standard deviation. Agglutination of *aca^-^* cells was compared to *aca^-^* cells expressing GFP-CAP (A) or GFP-N-Pro-CAP (B) and statistics were performed using the Student's t-test and the P value < 0.05 is indicated with *.

To address the mechanism mediating the aggregation of *aca^-^* cells expressing GFP-CAP or GFP-N-pro-CAP, we analyzed the cell adhesion systems and the requirement of Ca^2+ ^for contact formation. AX2 and AX2 cells expressing GFP-CAP showed the formation of agglutinates at 3 h that developed into large aggregates by 6 h in the absence of EDTA. Presence of 10 mM EDTA allowed agglutination at 6 h however the aggregates were smaller. The *aca^-^* cells remained as single cells in the presence or absence of EDTA (Figure 5). At 6 h after onset of starvation, *aca^-^* cells expressing GFP-CAP or GFP-N-pro-CAP showed aggregates in the absence of EDTA. However, in the presence of 10 mM EDTA cells were disaggregated (Figure 5). Next, we followed the time course of agglutination in the absence or presence of 10 mM EDTA in quantitative assays. AX2 and AX2 cells expressing GFP-CAP agglutinated in a comparable manner in the absence of EDTA. In the presence of EDTA agglutination was significantly delayed and strongly reduced. Cell-cell contacts became EDTA resistant at 6 h of starvation whereas in AX2 cells expressing GFP-CAP enhanced agglutination was observed earlier. The *aca^-^* cells expressing GFP-CAP or GFP-N-pro-CAP showed aggregate formation in the absence of EDTA, but in the presence of EDTA it was strongly reduced (Figure 6A, B). These results correlated with the microscopic images (Figure 5). Agglutination in *aca^-^* cells expressing GFP-CAP or GFP-N-pro-CAP was 1.5 fold greater in the absence of EDTA when compared to the presence of EDTA (Figure 6A, B). These data suggest that *aca^-^* cells expressing GFP-CAP or GFP-N-pro-CAP acquire the Ca^2+ ^dependent cell adhesion system active during early aggregation.

### GFP-CAP influences the expression of DdCAD1 to restore EDTA sensitive cell adhesion sites in *aca^-^*

**Figure 7:**
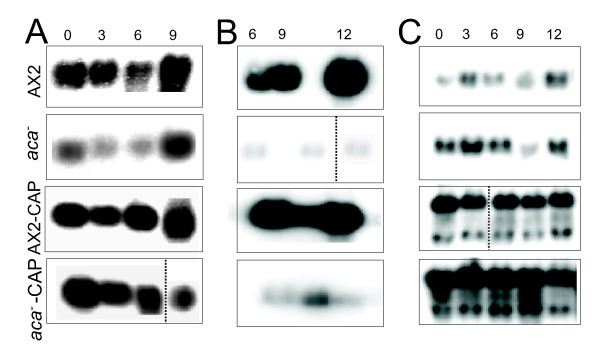
**Ectopic expression of GFP-CAP induces DdCAD1 mRNA levels in *aca^-^* cells**. Transcripts for the cell adhesion molecules DdCAD1 (A) and csA (B) were detected in AX2 and *aca^-^* cells and cells expressing GFP-CAP by northern blot analysis. 20 μg total RNA per time point (in hours, h) were used, probing was with DdCAD1 or csA cDNA probes. (C) Endogenous CAP and GFP-CAP mRNA levels were detected as controls. The increase in CAP mRNA amount in the *aca^-^* strain was not paralleled by an increase of the protein amounts (see Figure 1A and C, lanes 1, 2).

To further investigate the type of cell adhesion mechanism in *aca^-^* cell expressing GFP-CAP, we examined the expression of the cell adhesion molecules DdCAD1 and csA by determining their transcript levels in *aca^-^* cells and *aca^-^* expressing GFP-CAP. Northern blot analysis showed an increase in DdCAD1 transcripts in *aca^-^* cells expressing GFP-CAP in comparison to the *aca^-^* cells that showed very low expression at 3 and 6 h of starvation. The DdCAD1 transcript levels of AX2 and AX2 cells expressing GFP-CAP were comparable (Figure 7A). These results correlated with the enhanced agglutination noted for the *aca^-^* cells expressing GFP-CAP in the absence of EDTA. csA mRNA was seen in AX2 cells at the onset of aggregation and increased in amounts as described [23]. In AX2 expressing GFP-CAP the csA mRNA levels were strongly enhanced at the 6 h time point. The *aca^-^* cells showed dramatically reduced accumulation of the csA transcript with weak signals at every time point tested, whereas in *aca^-^* cells expressing GFP-CAP an increase was observed with a peak at 9 h (Figure 7B, 8A). Levels of CAP and GFP-CAP mRNA have been included for control (Figure 7C). Expression of cAMP receptor 1 (carA), a marker for early development, in *aca^-^* expressing GFP-CAP or GFP-N-pro-CAP resembled the pattern seen in AX2. In the *aca^-^* strong accumulation was slightly shifted to earlier time points (Additional file 7, Figure S7).

**Figure 8:**
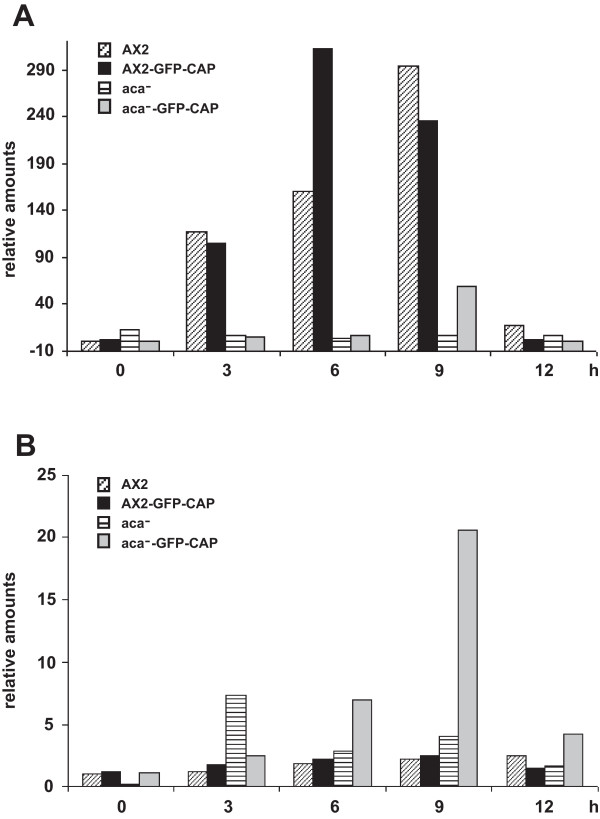
**csA and acrA transcript accumulation in wild type and mutant cells**. The expression of the csA (A) and acrA (B) gene was studied in the various strains by qRT-PCR using RNA from distinct developmental time points as indicated.

Taken together, these data suggest that expression of GFP-CAP influences the Ca^2+ ^dependent cell adhesion mechanism by mediating enhanced DdCAD1 expression. GFP-CAP expression enhanced the agglutination in AX2 cells both in the presence and absence of EDTA. We determined that at both 4 and 6 h after starvation, csA protein levels were higher in AX2 cells expressing GFP-CAP when compared to control AX2 (Figure 4A). Next, we analyzed the csA protein levels in *aca^-^* cells and transformants expressing GFP-CAP or GFP-N-pro-CAP. Immunoblots showed an 80 kDa protein corresponding to the csA protein in control AX2 and AX2 cells expressing GFP-CAP. csA protein was not detected in *aca^-^* cells and *aca^-^* cells expressing GFP-CAP or GFP-N-pro-CAP (Figure 4B), although EDTA resistant contact sites were restored to some degree upon expression of CAP and an increase in mRNA was noted (Figure 6, Figure 8A). These data suggest that expression of GFP-CAP influences csA expression and enhances formation of EDTA resistant cell adhesion sites in AX2, however it requires ACA to promote EDTA resistant cell adhesion as in *aca^-^* cells these contacts are strongly reduced.

As both the DdCAD1 and csA gene transcription is regulated by cAMP we asked whether the adenylyl cyclase ACB which is encoded by the acrA gene and is present throughout all developmental stages is responsible for supplying endogenous cAMP and tested the levels of acrA mRNA by quantitative PCR (qRT-PCR) [5,13]. We found that in *aca^-^* the amount of acrA mRNA was increased as compared to AX2 and that in *aca^-^* expressing GFP-CAP expression acrA mRNA levels were further enhanced (Figure 8B).

## Discussion

Many important biological processes including cell adhesion and development are mediated through an array of signaling proteins and pathways [5]. Our study showed that CAP, an important regulator of cell polarity, determines aggregation, cell adhesion and development in *D. discoideum*. Interestingly, expression of CAP in *aca^-^* cells led to the restoration of cell polarity, streaming and aggregation defects. Although expression of CAP restored the severe aggregation defects, the cells failed to complete the developmental process and did not form multicellular fruiting bodies suggesting the requirement of ACA and associated signaling for this process. The role of CAP in influencing the expression of cell adhesion molecules DdCAD1 and csA that mediate calcium-dependent and -independent cell adhesion, respectively, suggested that CAP acts at the intersection of signaling to link cell polarization to aggregation, cell adhesion and multicellular development in *D. discoideum*.

*D. discoideum *has developed a remarkable versatile mechanism for pulsatile synthesis and secretion of cAMP that is the backbone of its capacity for self-organization. ACA is responsible for the production of extracellular cAMP and the relay response [22,24]. In the *D. discoideum *CAP mutant CAP bsr, the cAMP relay response and ACA activity after GTPγS stimulation was much lower suggesting the requirement of CAP for the cAMP relay. Furthermore, ACA expression was reduced in unstimulated CAP bsr cells [18]. Our present study revealed that levels of endogenous CAP and ectopic expression of CAP or its domains fused to GFP remained unaltered in unstimulated *aca^-^* cells suggesting that ACA does not influence CAP expression. Also, the localization and redistribution of CAP did not require ACA for proper targeting and transient redistribution of CAP during macropinocytosis or phagocytosis, thereby suggesting a functioning of CAP independent of ACA. In yeast, the proline rich domain of CAP that binds to SH3-domain containing proteins is responsible for the proper localization of CAP at the cortical cytoskeleton [25,26]. In contrast to yeast, the N-terminus of CAP mediates the correct localization in *D. discoideum *[17].

Previously, we have shown that localization, redistribution and functioning of CAP acts in a G-protein independent pathway and requires neither the cAMP receptor nor PI3 kinase but rather depends on the activity of protein kinase A (PKA), an essential regulator for all stages of *D. discoideum *development [19]. Furthermore, it has been suggested that during *D. discoideum *development, all intracellular signaling by cAMP is mediated by the cAMP dependent protein kinase PKA, since cells carrying null mutations in the *aca *gene can develop so as to form fruiting bodies under some conditions if PKA is constitutively active by overexpressing the catalytic subunit [27,28]. Also, it has been shown that ACA is not required to produce intracellular cAMP for PKA activation but is essential for the production of extracellular cAMP and coordination of cell movement during all steps of development and for induction of developmental gene expression [24]. However, later it has been revealed that cells lacking both ACA and ACB adenylyl cyclases develop and form mounds at higher cell densities, express cell specific developmental genes at reduced levels and secrete cellulose coats but do not form fruiting bodies even when PKA is constitutively expressed which indicated that synthesis of cAMP is required for spore differentiation [13]. Our data suggested that CAP plays an important role in cell polarization, streaming and aggregation but still requires the ACA activity or ACA associated signal transduction pathways for the production of extracellular cAMP to complete development and fruiting body formation.

A role for a cytoskeletal component in regulating chemotactic signaling has been recently reported for *D. discoideum *cells expressing mutant actin Y53A which can not get phosphorylated on tyrosine 53. Expression of this protein caused a reduction of cell surface cAMP receptors, inhibited cAMP-induced increases in adenylyl cyclase A activity and further events. It was proposed that altered cAMP signaling may be due to a disorganized cytoskeleton [29]. Additional work from the same group supports this mechanism. They showed that the actin crosslinking proteins cortexillin I and II are required for cAMP signaling during chemotaxis and development as they have an effect on adenylyl cyclase synthesis and activity [30]. These results indicate that a complex network of cytoskeletal proteins is acting in this process. How and whether they influence each other is not known. For plant CAP an involvement in signaling pathways has been demonstrated as well [31].

*D. discoideum *amoebae depend on chemoattractant stimulation to acquire polarity, a prerequisite for the development of cell migration [15,32]. Additionally, cGMP production and signaling from cell surface receptors to chemotactically induced cell polarization and pseudopod formation has been shown to influence myosin recruitment to the actin cytoskeleton in order to attain an elongated polarized morphology [33]. The altered cAMP relay response and reduced sensitivity to outside chemotactic signals in CAP bsr cells contributed to their poor polarization behavior. Moreover, regulation of myosin assembly was disorganized, which resulted in additional formation of pseudopods in aggregation competent CAP bsr cells, suggesting CAP as an important regulator of cell polarity [18]. Kriebel et al. [14] have shown that during polarization, ACA-YFP was highly enriched at the uropod of polarized chemotaxing cells and this localization of ACA was independent of the regulator CRAC (cytosolic regulator of adenylyl cyclase) and the effector PKA. Interestingly, it was found that the asymmetric distribution of ACA-YFP was dependent on the actin cytoskeleton and on the acquisition of cellular polarity and its regulators. The actin-based cytoskeleton is a primary regulator of cell shape, polarity and movement in the eukaryotic cells. We found that ectopic expression of GFP-CAP restored cellular polarity and led to normal distribution of the polarity markers myosin II, α-actinin and filamin in *aca^-^* cells, suggesting an important role for CAP in acquiring cellular polarity by proper organization of the actin-myosin complex. Cells lacking ACA were capable of moving up the chemoattractant gradient but were unable to polarize, stream and orient themselves in a head to tail fashion as they migrate to form aggregates [14]. Ectopic expression of GFP-CAP restored the polarity, streaming and aggregation defects of *aca^-^* cells suggesting a function of CAP downstream of ACA in attaining cellular polarity. Furthermore, expression of GFP-N-pro-CAP was efficient to restore these defects in *aca^-^* cells. This data suggested an important role for the N-terminus of CAP in cell polarity, cAMP signaling, chemotactic migration and development.

Previously, it has been shown that accumulation of CAP in actin-rich regions at moving fronts favors polarization and it was suggested to be a function of the actin binding domain in the CAP C-terminus [16,17]. It has also been suggested that both the C- and N-terminal domains of CAP independently affect F-actin polymerization, whereby the C-terminus directly interacts with F-actin and the N-domain interacts with an actin-cofilin complex thereby indirectly influencing the actin cytoskeleton during polarization [34]. Our studies show that the N-terminus of CAP favors cell polarization and corroborates previous studies which have suggested that the N-terminus of yeast CAP, an interaction domain for adenylyl cyclase, mediates correct signaling activities during polarization [20].

Our data suggest that CAP plays an important role in the regulation of cell adhesion molecules by influencing the expression of DdCAD1 and csA. Expression of genes involved in aggregation and post-aggregation can be induced by the addition of extracellular cAMP, suggesting that loss of cell signaling rather than a requirement for cell-cell adhesion is responsible for the absence of such developmentally regulated genes [35]. In fact, DdCAD1 and csA harbor cAMP responsive elements in their promoters and depend on this hormone, and it might be this signaling pathway which involves CAP [36]. In addition, DdCAD1 expression is controlled by prestarvation factor, PSF, and csA is induced in the early aggregation stage by a cAMP-independent mechanism [37].

Interestingly, ectopic expression of CAP, a regulator of the cAMP relay response, enhanced the DdCAD1 and csA mRNA levels. Our data show that DdCAD1 mRNA levels were dramatically reduced during aggregation (3-6 h) in *aca^-^* cells and expression of GFP-CAP restores the expression of DdCAD1, thereby reversing the severe defects in streaming, aggregation and EDTA sensitive cell adhesion of *aca^-^* cells. Ectopic expression of CAP restored the EDTA sensitive-DdCAD1 mediated cell adhesion but failed to improve the EDTA resistant csA mediated cell adhesion. This failure could be due to the reduced levels of csA and inefficiency to maintain the stability of the cell-cell contacts and incapability to retain DdCAD1 in cell-cell contacts of post-aggregation stage *aca^-^* cells expressing GFP-CAP. Expression of GFP-CAP promoted the early expression of csA protein at 4 and 6 h during the development of AX2, whereas it failed to significantly increase the expression of csA in *aca^-^* cells. Both, the reduced mRNA and protein levels of csA correlated with the severe defects in aggregation and development of *aca^-^* cells. Loss of csA expression is shown to result in increased cell-substratum adhesion and reduced motility, aggregation and cell-cell adhesion [6,38]. GFP-CAP expression enhanced streaming in *aca^-^* cells but this reversion was independent of high csA levels. It is unlikely that csA overexpression directly induces development as a mutant that lacks csA develops wild-type-looking fruiting bodies under normal conditions, however, only a few fruiting bodies formed viable spores. Also, cells overexpressing csA exhibited a degree of aberrant multicellular development (larger slugs and bigger fruiting bodies), but that was related to excessive adhesion rather than aberrant gene expression [36,38,39]. The second EDTA-resistant cell binding system mediated by the glycoprotein gp150 encoded by the *lagC *gene is expressed at low levels in the mid-aggregation stage, followed by a rapid increase that coincides with the completion of aggregate formation [40]. We have not analyzed the expression of gp150 in *aca^-^* cells and *aca^-^* cells expressing GFP-CAP, because in contrast to aggregation stage genes, *lagC *transcription is not affected by nanomolar pulses of cAMP, but requires high levels of cAMP in the post aggregation stage [5,10].

In summary, cell-cell adhesion is important for morphogenesis and tight regulation of gene expression in the multicellular context. Dysfunction of cell adhesion molecules often leads to diseases and abnormalities in fetal development. *D. discoideum *cell-cell adhesion involves several processes common to the metazoa, and numerous proteins that regulate *D. discoideum *cell-cell adhesion, and signaling sharing both sequence and functional similarity to their counterparts in mammals. Our data revealed a novel role for CAP in aggregation and cell-cell adhesion that embarks on a journey of future discovery and studies related to mammalian wound healing and closure of major accidental injuries.

## Conclusions

CAP affects the F-actin/G-actin ratio and is a regulator of cell polarity. Ectopic expression in cells lacking the aggregation stage specific adenylyl cyclase A rescues the cell polarity defect in these cells and allows cell aggregation. The underlying mechanism is an induction of expression of cell adhesion molecules which are required for multicellular development.

## Methods

### Strains and reagents

*D. discoideum *wild type strain AX2, aggregation-specific adenylyl cyclase mutant (*aca^-^*) and *aca^-^* transformants expressing full length CAP or its domains used in this study were cultured at 21°C as described [41]. For rescue experiments, *aca^-^* cells were transformed with vectors allowing for the expression of GFP fusions of full length CAP, N- or C-terminal domains with or without the proline rich-regions under the control of the constitutively active actin15 promoter. Specifically, we used the N-terminal domain of CAP (GFP-N-CAP, amino acid residues 1 to 215) and the N-terminus including the proline rich region (GFP-N-pro-CAP, amino acids 1-254). For the C-terminal domain fusions with (GFP-pro-C-CAP, residues 216-464) or without the proline rich region (GFP-C-CAP, amino acids 255-464) were used. Constructions of these GFP fusions have been described previously; the GFP tag was at the C terminus of the fusion proteins [17]. Expression of CAP and its domains was nearly identical to the levels of wild type protein as assessed by Western blot analysis using CAP domain-specific mAbs 230-18-8 or 223-445-1 against the N- or C-terminal domains, respectively [16]. Contact site A antibody (33-294-17; [42]) was used to monitor the developmental stages. Recombinant GFP was recognized with mAb K3-184-2, that was raised against wild type GFP [18]. Monoclonal antibodies specific to myosin (56-395-2), α-actinin (47-62-2) and filamin (82-454-12) were used in the immunofluorescence assays [43,44]. Actin was detected with mAb act1 [45]. All these primary antibodies were generated as hybridoma supernatants in our laboratory. Cy3 labeled, or horseradish peroxidase conjugated goat anti-mouse IgG secondary antibodies (Sigma) were used for detection. Examination was done with a confocal microscope (Leica TCS SP5).

### Growth and development of *D. discoideum*

Wild type AX2, *aca^-^* cells and the derived transformants were grown in axenic liquid medium containing appropriate antibiotics such as G418 (4 μg/ml) depending on the strains, either in shaking suspension (160 rpm) or in petri dishes at 21°C [41]. For developmental studies, exponentially growing cells were harvested from liquid medium, washed twice in Soerensen phosphate buffer (17 mM Na^+^/K^+^-phosphate buffer, pH 6.0) and continued shaking for the indicated times or alternatively plated onto SM agar plates overlaid with *Klebsiella aerogenes *and incubated at 21°C for 3-4 days until *D. discoideum *plaques appeared on the bacterial lawns. Single plaques were picked up with sterile toothpicks and transferred either to new *Klebsiella *overlaid SM agar plates or resuspended in liquid medium with selective antibiotics. Developmental phenotypes were noted and imaged at indicated time points. Transformation was carried out as described [19].

### Microscopy and live cell imaging

Axenically grown cells were harvested at densities of 1 × 10^6 ^cells/ml and allowed to adhere onto 18 mm acid-washed glass coverslips for 30-60 min. Cells were either fixed with methanol for 10 min (-20°C) or with picric acid/paraformaldehyde for 20 min (room temperature) as described [41]. Distribution of GFP-CAP was analyzed during phagocytosis and fluid phase endocytosis as described [19,46]. Briefly, adherent cells were incubated with 300 μl of Soerensen phosphate buffer containing heat killed unlabeled yeast cells (for phagocytosis) or with 2 mg/ml of TRITC-dextran (for pinocytosis), followed by fixation with methanol to analyze phagocytosis or with paraformaldehyde/picric acid to examine macropinocytosis. For live cell imaging, confocal images obtained at different times were assembled at equal and optimized averaging and a sectioning of 200 nm in the Leica confocal software. For developmental studies, 1 × 10^7 ^cells/ml were starved for 6 h in Soerensen phosphate buffer at 21°C, harvested and washed twice and allowed to adhere onto glass coverslips for 15-30 min before fixation with methanol (-20°C). During starvation and at later developmental stages, phase contrast images were obtained at indicated times using 20 × bright field phase contrast microscopy (Olympus).

### Endocytosis Assays

Fluid-phase endocytosis and phagocytosis assays were performed as described [19,41]. Briefly, growing cells at densities < 5 × 10^6 ^cells/ml were centrifuged and resuspended at 2 × 10^6 ^cells/ml in fresh axenic medium and incubated at 21°C, 160 rpm, for 15 min to recuperate. Cells were incubated either with TRITC-dextran (2 mg/ml) for fluid-phase endocytosis or TRITC-labeled yeast (10^9 ^yeast cells/ml) for phagocytosis assays. Samples were collected at different intervals and the fluorescence of non-specifically bound TRITC marker or non-internalized yeast cells were quenched with trypan blue (2 mg/ml) by incubating for 3 min. Cells were centrifuged, resuspended in Soerensen phosphate buffer and fluorescence was measured using a fluorimeter (544 nm excitation/574 nm emission).

### Agglutination assays

Axenically growing AX2, *aca^-^* cells or respective transformants expressing GFP-CAP or GFP fusions of CAP domains were harvested, washed twice and resuspended in Soerensen phosphate buffer to a density of 1 × 10^7 ^cells/ml and incubated at 160 rpm at 21°C. During starvation, samples were collected at indicated time points and the decrease in light scattering was measured at 600 nm. For determining EDTA sensitive or resistant cell adhesion [47], samples were starved in suspension for the desired periods of time and incubated for an additional hour either in the presence or absence of 10 mM EDTA. The decrease in light scattering was measured at 600 nm.

### Isolation of total RNA from *D. discoideum *cells and quantitative Real Time PCR (qRT-PCR)

Axenically growing or aggregation competent cells (6 h of starvation) were harvested (1 × 10^8 ^cells), washed twice with ice-cold DEPC treated H_2_O (0.1% DEPC, mixed by stirring for 5-6 h, autoclaved) and lysed in 1 ml of RLT buffer. RNA was extracted using Qiagen RNeasy kit following the manufacturer's instructions. RNA was quantified by measuring the OD_260 _and stored at -80°C until use. RNA gel electrophoresis and northern blot analysis were carried out as described [17].

For qRT-PCR quantity and quality of RNA was analysed on an Agilent Bioanalyser (Agilent Technologies). cDNA was prepared by reverse transcription of 5 μg RNA with oligo dT using Superscript II reverse transcriptase (Invitrogen). Real time PCR was carried out with the Opticon III instrument (MJ Research) using the Quantitect™ SYBR^® ^green PCR kit (Qiagen, Hilden, Germany) according to [19]. As a quantification standard defined concentrations of annexinA7 cDNA [48] were used for amplification. For every cDNA quantification three reactions were performed in parallel.

### SDS-PAGE and Immunoblotting

SDS-polyacrylamide gel electrophoresis was performed using the discontinuous buffer system. Proteins were resolved on 10-15% resolving and 5% stacking gels and after electrophoresis proteins were transferred to nitrocellulose membranes using a semi-dry blotter (Biorad). Blots were blocked overnight with 5% milk powder at 4°C, followed by incubations with primary and secondary antibodies for 1 h each at room temperature. After incubation with antibodies, blots were thoroughly washed and processed for enhanced chemiluminescence (ECL).

## Authors' contributions

HS designed and carried out the experiments, analyzed the data and wrote the manuscript, GN designed and carried out experiments and analyzed the data, FR designed experiments and analyzed data, RBW carried out experiments and analyzed data, MS analyzed data, AAN designed the experiments, analyzed the data and wrote the manuscript. All authors read and approved the final manuscript.

## Supplementary Material

Additional file 1 **Figure S1. Distribution of GFP-CAP or its domains is unaltered in *aca^-^* cells**. The *aca^-^*cells expressing the GFP fusions of full length CAP or its N- or C-domains with or without the proline rich regions were fixed with methanol at -20°C for 10 min and immunolabeled with CAP specific mAb 230-18-8 which recognizes the GFP fusions of full length CAP, N-domain fusions and endogenous protein. In *aca^-^*cells, GFP-CAP distributed within the cytosol and was enriched at the cortex. The N-terminal fusions of CAP showed localization throughout the cytoplasm with enrichments at the cell periphery. The C-terminal GFP fusions showed distribution throughout the cytosol and no enrichments at the regions close to the membrane. For comparison we show the localization of full length CAP in AX2 cells (lowest panel). We have chosen a cell which expresses GFP-CAP at low levels allowing easy evaluation of the distribution. Bar, 10 μm. Click here for file

Additional file 2 **Figure S2. Redistribution of GFP-CAP or GFP-N-CAP during macropinocytosis is unaffected in *aca^-^*cells**. (A) Live dynamics of GFP-CAP during macropinocytosis of *aca^-^*cells. Cells were allowed to adhere on coverslips and challenged with TRITC dextran (marker for pinocytosis) and series of images were obtained with confocal microscopy. GFP-CAP redistributed from the cytosol and cell periphery to regions forming macropinocytic cups and macropinosomes. Images were collected at indicated times (in seconds) and arrowheads denote enrichment of GFP-CAP to the regions of interest. (B) Distribution of GFP fusions of CAP or N-CAP in *aca^-^*cells fixed during macropinocytosis. Arrowheads point at macropinocytic cups. The localization of full length CAP during macropinocytosis in AX2 cells is shown in the bottom panel. Arrowheads point at macropinocytic cups. Bar, 10 μm. Click here for file

Additional file 3 **Figure S3. Redistribution GFP-CAP proteins during phagocytosis is unaltered in the absence of ACA**. (A) *aca^-^*cells and transformants expressing GFP-CAP or its domains were allowed to adhere onto coverslips and were exposed to unlabelled yeast cells for 15 min, fixed with methanol (-20°C) for 10 min and immunostained with CAP specific mAb 230-18-8. The distribution of full length GFP-CAP in AX2 is shown for comparison. Bar, 10 μm. (B) Shown are the quantitative analysis of phagocytosis in *aca^-^*cells expressing GFP fusions of CAP or its domains. 2 × 10^6 ^cells were treated with TRITC labeled heat killed yeast cells and fluorescence of non-internalized yeast was quenched with trypan blue. Fluorescence from internalized yeast was measured at the indicated time points. Data are represented as relative fluorescence. Bars represent the standard deviation from the average value of three independent experiments. Click here for file

Additional file 4 **Figure S4. Localization of myosin II, α-actinin and filamin in *aca^-^* cells**. Aggregation competent AX2 and *aca* cells were starved for 6 h, fixed with methanol at -20°C and immunostained with antibodies specific to myosin (56-395-2), α-actinin (47-62-2) or filamin (82-454-12). Confocal microscopy showed myosin at posterior and lateral cell projections, α-actinin localized to the cytosol and was enriched in pseudopods, and filamin distributed at the posterior and the sides in AX2 cells. AX2 cells were highly polarized and showed elongated shapes in comparison to *aca^- ^*cells. However, similar localization patterns were observed, i.e. cortical localization for myosin and filamin and cytosolic distribution for α-actinin. Bar, 10 μm. Click here for file

Additional file 5 **Figure S5. Localization of GFP-CAP in AX2 cells**. Aggregation competent AX2 cells expressing GFP-CAP were starved for 6 h, fixed with methanol at -20°C and immunostained with antibodies specific to myosin (56-395-2), α-actinin (47-62-2) and filamin (82-454-12). Confocal microscopy showed that GFP-CAP in AX2 cells colocalizes with myosin, α-actinin and filamin at cortical regions. Insets show single cells taken from different microscopic fields. Bar, 10 μm. Click here for file

Additional file 6 **Figure S6**. **Expression of GFP-CAP or GFP-N-pro-CAP influenced the polarity defects of *aca^-^* cells**. Aggregation competent *aca^-^* cells expressing GFP-CAP (A) or GFP-N-pro-CAP (B) were starved for 6 h, fixed with methanol at -20°C and immunostained with antibodies specific to myosin (56-395-2), α-actinin (47-62-2) and filamin (82-454-12). Confocal microscopy showed that GFP-CAP (A) and GFP-N-pro-CAP (B) in *aca^-^* cells colocalize with myosin, α-actinin and filamin at cortical regions. Insets show single cells taken from different microscopic fields. Bar, 10 μm. Click here for file

Additional file 7 **Figure S7**. **Analysis of cAR1 expression in *aca^-^* cells expressing GFP-CAP or GFP-N-pro-CAP**. Northern blot showing the levels of cAR1 mRNA were unaltered in *aca^-^* cells and cells expressing GFP-CAP or GFP-N-pro-CAP. AX2 served as control. The time of development is given in hours (h). Click here for file
