# Supplementary figures and images for: Ectopic expression of cyclase associated protein CAP restores the streaming and aggregation defects of adenylyl cyclase a deficient Dictyostelium discoideum cells
Source: BMC Dev Biol. 2012 Jan 12;12:3. doi: 10.1186/1471-213X-12-3 (PMC3316131; doi:10.1186/1471-213X-12-3)

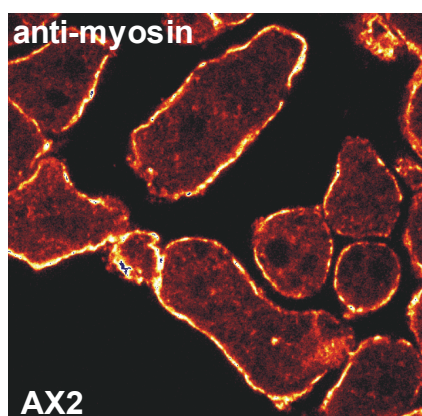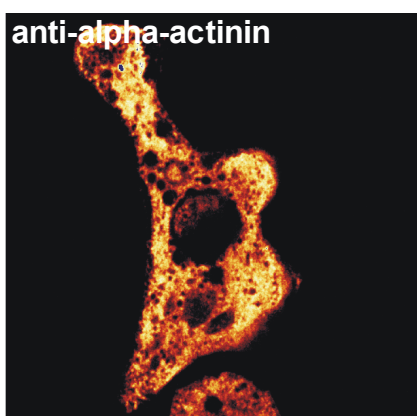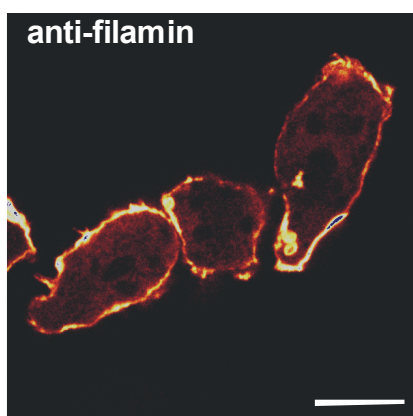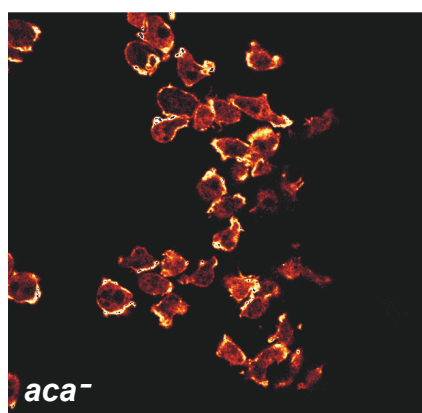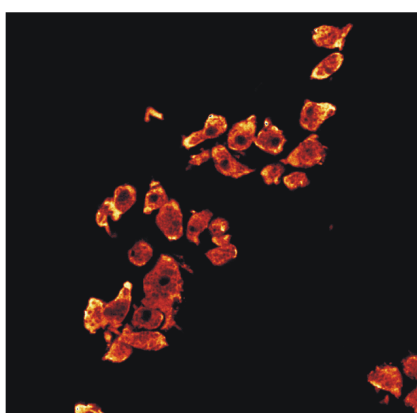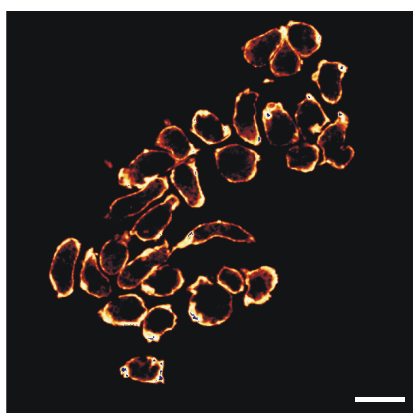

Supplement: Additional file 4 — Figure S4. Localization of myosin II, α-actinin and filamin in aca- cells. Aggregation competent AX2 and aca cells were starved for 6 h, fixed with methanol at -20°C and immunostained with antibodies specific to myosin (56-395-2), α-actinin (47-62-2) or filamin (82-454-12). Confocal microscopy showed myosin at posterior and lateral cell projections, α-actinin localized to the cytosol and was enriched in pseudopods, and filamin distributed at the posterior and the sides in AX2 cells. AX2 cells were highly polarized and showed elongated shapes in comparison to aca- cells. However, similar localization patterns were observed, i.e. cortical localization for myosin and filamin and cytosolic distribution for α-actinin. Bar, 10 μm. [file 1471-213X-12-3-S4.PDF]

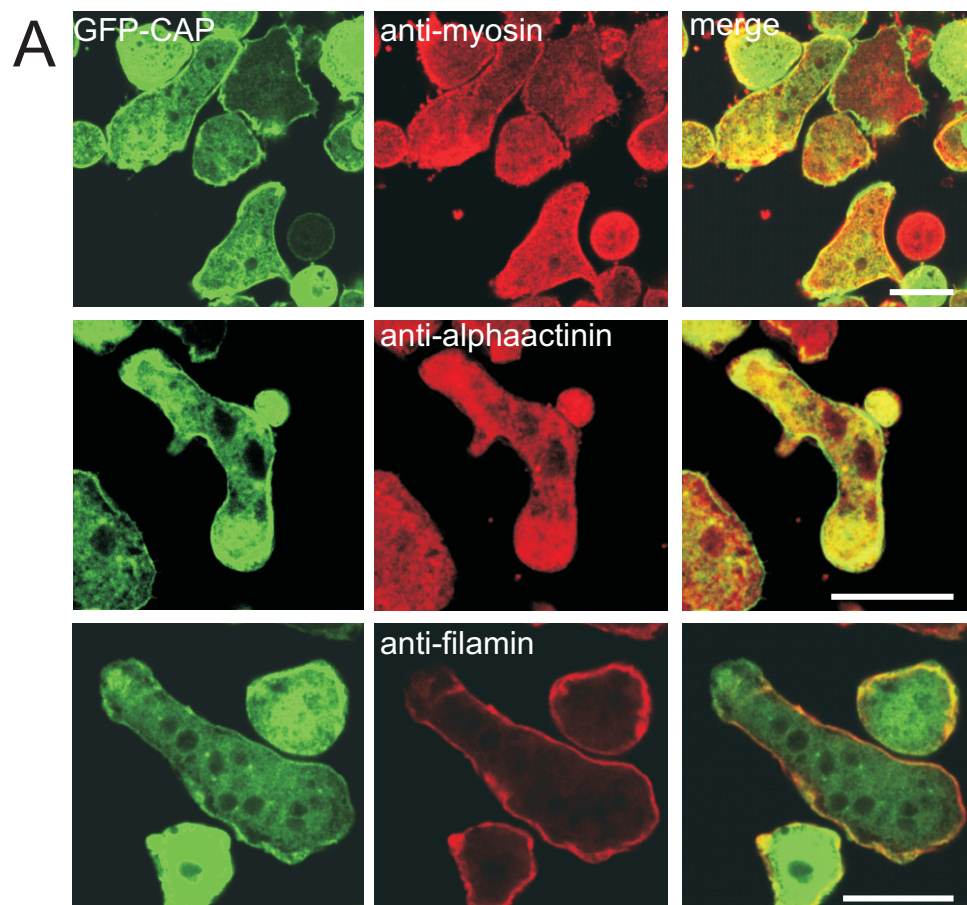

Supplement: Additional file 5 — Figure S5. Localization of GFP-CAP in AX2 cells. Aggregation competent AX2 cells expressing GFP-CAP were starved for 6 h, fixed with methanol at -20°C and immunostained with antibodies specific to myosin (56-395-2), α-actinin (47-62-2) and filamin (82-454-12). Confocal microscopy showed that GFP-CAP in AX2 cells colocalizes with myosin, α-actinin and filamin at cortical regions. Insets show single cells taken from different microscopic fields. Bar, 10 μm. [file 1471-213X-12-3-S5.PDF]

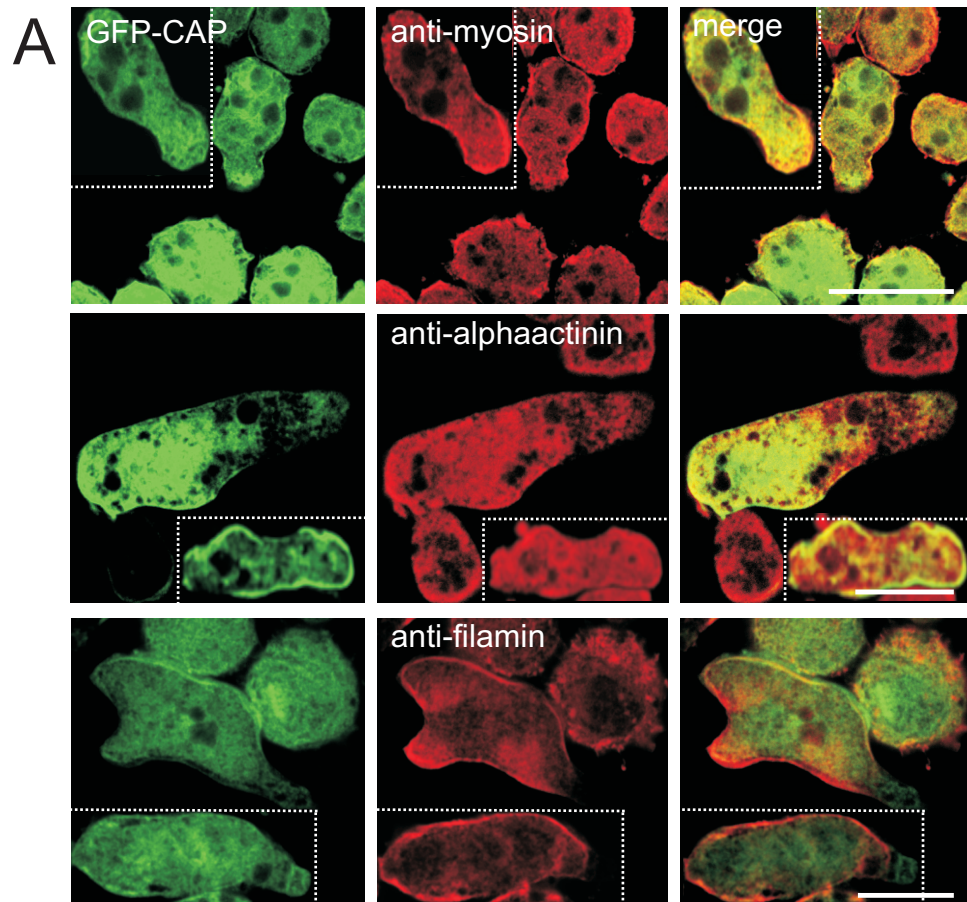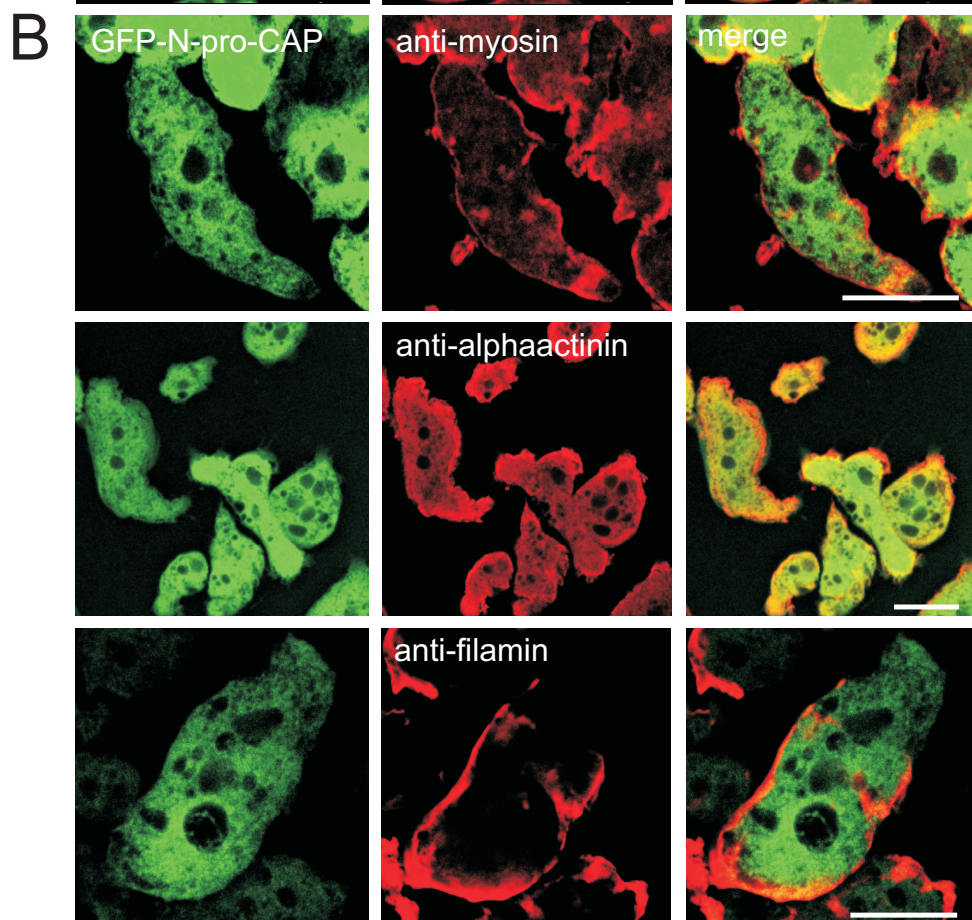

Supplement: Additional file 6 — Figure S6. Expression of GFP-CAP or GFP-N-pro-CAP influenced the polarity defects of aca- cells. Aggregation competent aca- cells expressing GFP-CAP (A) or GFP-N-pro-CAP (B) were starved for 6 h, fixed with methanol at -20°C and immunostained with antibodies specific to myosin (56-395-2), α-actinin (47-62-2) and filamin (82-454-12). Confocal microscopy showed that GFP-CAP (A) and GFP-N-pro-CAP (B) in aca- cells colocalize with myosin, α-actinin and filamin at cortical regions. Insets show single cells taken from different microscopic fields. Bar, 10 μm. [file 1471-213X-12-3-S6.PDF]

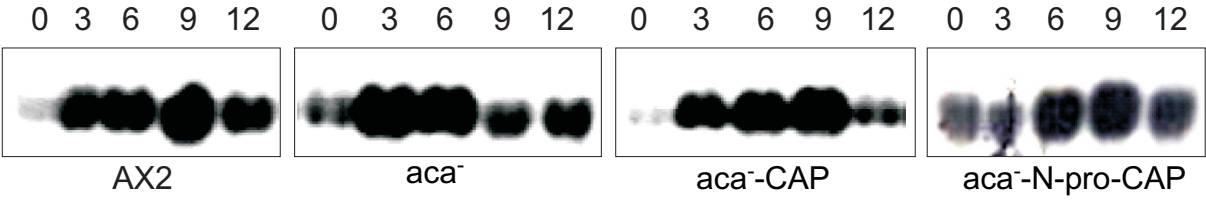

Supplement: Additional file 7 — Figure S7. Analysis of cAR1 expression in aca- cells expressing GFP-CAP or GFP-N-pro-CAP. Northern blot showing the levels of cAR1 mRNA were unaltered in aca- cells and cells expressing GFP-CAP or GFP-N-pro-CAP. AX2 served as control. The time of development is given in hours (h). [file 1471-213X-12-3-S7.PDF]
